# Expectations and Challenges of First-Line Maintenance Therapy for Advanced Ovarian Cancer

**DOI:** 10.3390/medicina57050501

**Published:** 2021-05-15

**Authors:** Tadahiro Shoji, Chie Sato, Hidetoshi Tomabechi, Eriko Takatori, Yoshitaka Kaido, Takayuki Nagasawa, Masahiro Kagabu, Tsukasa Baba

**Affiliations:** Department Obstetrics and Gynecology, Iwate Medical University School of Medicine, Iwate 028-3695, Japan; c.kurokawa0825@gmail.com (C.S.); bechitomabehi@gmail.com (H.T.); sppe8459@yahoo.co.jp (E.T.); kaido0428@yahoo.co.jp (Y.K.); shirokuma723@ybb.ne.jp (T.N.); m.kagabu@nifty.com (M.K.); babatsu@iwate-med.ac.jp (T.B.)

**Keywords:** ovarian cancer, maintenance therapy, bevacizumab, PARP inhibitor, immune checkpoint inhibitor

## Abstract

The incidence of ovarian cancer, which has had a poor prognosis, is increasing annually. Currently, the prognosis is expected to improve with the use of molecular-targeted drugs and immune checkpoint inhibitors as maintenance therapies after the first-line chemotherapy. The GOG218 and ICON7 studies reported the usefulness of bevacizumab and the SOLO-1 and PRIMA (A Phase 3, Randomized, Double-Blind, Placebo-Controlled, Multicenter Study of Niraparib Maintenance Treatment in Patients With Advanced Ovarian Cancer Following Response on Front-Line Platinum-Based Chemotherapy) studies have reported the usefulness of olaparib and niraparib, respectively. The ATHENA study investigating the usefulness of rucaparib is currently ongoing. Although clinical studies of immune checkpoint inhibitors are lagging in the field of gynecology, many clinical studies using programmed death cell-1 (PD-1) and PD-1 ligand 1 (PD-L1) antibodies are currently ongoing. Some biomarkers have been identified for molecular-targeted drugs, but none have been identified for immune checkpoint inhibitors, which is a challenge that should be addressed in the future.

## 1. Introduction

The incidence of ovarian cancer is increasing every year and is one of the most common gynecological malignancies, ranking third after cervical and uterine cancer. In 2017, there were 22,440 estimated new diagnoses of ovarian cancer and 14,080 deaths from the disease in the USA; deaths were higher than from endometrial and cervical cancer [1].

Ovarian cancer often progresses without symptoms at an early stage and, consequently, 60%–70% of all newly diagnosed patients are at advanced stages III to IV. Based on the histological type of the cancer, clear cell and mucinous adenocarcinomas are less sensitive to chemotherapy than serous and endometrioid adenocarcinomas [2,3]. Furthermore, the frequencies of clear cell and mucinous adenocarcinomas are higher in Japan than in Europe and the US [4]. In addition, the recurrence rate of serous adenocarcinoma with high sensitivity to chemotherapy is not low, which is a future challenge.

Overall survival (OS) did not improve in any of the GOG178 [5], AGO-GINECO [6], MITO-1 [7], or After-6 [8] studies, which examined the usefulness of maintenance therapy after the first-line chemotherapy in improving the prognosis of ovarian cancer. Consequently, maintenance therapy was considered to be ineffective (Table 1). However, the prognosis is expected to improve with the use of molecular-targeted drugs and immune checkpoint inhibitors as maintenance therapy instead of cytotoxic anticancer drugs. Recently, maintenance therapy with molecular-targeted agents following the first-line chemotherapy has drawn attention. In addition, immune checkpoint inhibitors are drawing attention as new drugs that show high efficacy in the treatment of solid cancers. However, the use of these agents in clinical practice is relatively new; therefore, it is necessary to accumulate additional data on currently unknown adverse reactions and strategies to treat them. It has been pointed out that the response rate to immune checkpoint inhibitors is lower than that of chemotherapy because biomarkers have not been established for many drugs.

In this article, we will provide an introductory review of the characteristics of maintenance therapy with molecular-targeted drugs and immune checkpoint inhibitors after the first-line chemotherapy for ovarian cancer, based on the results of clinical studies. In addition, we will discuss the respective challenges and actions of these agents.

## 2. Current Status of First-Line Chemotherapy for Ovarian Cancer

In the first-line chemotherapy for ovarian cancer, conventional paclitaxel plus carboplatin (TC) therapy, in which paclitaxel and carboplatin are administered every 3 to 4 weeks, is considered the standard therapy worldwide [9]. A regimen that has improved survival compared to this TC therapy is dose-dense TC (dd-TC) therapy, where the dosing interval of paclitaxel (every 3–4 weeks) is reduced to weekly dosing. This dd-TC therapy was investigated by the Japanese Gynecologic Oncology Group (JGOG) in the JGOG3016 phase 3 study that compared the usefulness of dd-TC and TC therapies in 631 patients with stage II–IV epithelial ovarian, fallopian tube, or peritoneal cancer [10]. In the final data, the median PFS rates at a 76.8 month follow-up were 28.2 months and 17.5 months in the dd-TC and TC groups, respectively, indicating a significant prolongation in the dd-TC group compared to the TC group (hazard ratio (HR) = 0.76). The median OS rates were 100.5 months and 62.2 months, respectively, indicating a prolongation in the dd-TC group compared to the TC group (HR = 0.79) [11].

Subsequently, in response to the results of the JGOG3016 study, the GOG262 study by the US Gynecologic Oncology Group (GOG) [12] and the ICON8 study by the International Collaborative Ovarian Neoplasm (ICON) group in Europe [13] were conducted (Table 2). The GOG262 study compared the usefulness of dd-TC with that of TC therapy in 692 patients with stage II–IV epithelial ovarian, fallopian tube, or peritoneal cancer. In this study, bevacizumab was used concomitantly and for maintenance (concomitant use rate: 84%). However, the results did not support the usefulness of dd-TC therapy [12], and dd-TC co-therapy with bevacizumab is not recommended. It can also be interpreted that the usefulness of dd-TC therapy is unknown when bevacizumab is not used concomitantly. The ICON8 study—conducted with 1566 patients with stage IC–IV epithelial ovarian, fallopian tube, or peritoneal cancer—compared the usefulness of three regimens of TC therapy, dd-TC therapy, and weekly TC therapy, where the dosing interval of carboplatin in the dd-TC therapy (every 3 weeks) was reduced to weekly dosing. Bevacizumab was not used concomitantly in this study, and the protocol was similar to that of the JGOG3016 study. However, the results did not demonstrate the usefulness of dd-TC therapy without bevacizumab [13]. Racial differences have been identified as one of the reasons why the ICON8 study did not demonstrate the usefulness of dd-TC shown in the JGOG3016 study. As described above, clear cell adenocarcinoma accounts for the majority of ovarian cancer cases in Japan, and it has been suggested that the therapeutic effect and the occurrence of toxicity may be affected by differences in single nucleotide polymorphisms, as shown in the Japan–US comparison of patients with small-cell lung cancer who were receiving TC therapy [14].

## 3. Maintenance Therapy with Molecular-Targeted Drugs

Molecular-targeted drugs include angiogenesis inhibitors and poly ADP-ribose polymerase (PARP) inhibitors, which each have different mechanisms of action. Angiogenesis inhibitors act on vascular endothelial growth factor (VEGF), which is required for the formation of blood vessels that deliver nutrients and oxygen to cancer cells, to inhibit cancer growth and proliferation. In contrast, PARP inhibitors inhibit the action of PARP, which is necessary for the repair of DNA damage and suppresses the proliferation of cancer cells. Recently, clinical studies have demonstrated prolongation of PFS with the use of these drugs as maintenance therapy after the first-line chemotherapy [15,16,17,18,19] (Table 3). These clinical studies with the indicated agents are described below.

### 3.1. Bevacizumab

Bevacizumab, an antibody against VEGF, was approved for coverage by health insurance in November 2013 as a molecular-targeted drug for ovarian cancer in Japan. Phase 3 clinical studies investigating the add-on effect of bevacizumab on TC therapy as the first-line of chemotherapy include the GOG218 [15] and ICON7 [16] studies. In the GOG218 study, bevacizumab (15 mg/kg) was administered for 21 cycles (from cycles 2 to 22 of TC therapy) to 1873 patients with high-risk stage III (residual disease after primary cytoreduction) and IV ovarian cancer after primary debulking surgery, and the results were compared with those of the TC plus placebo group. The median PFS was 10.3 months and 14.1 months in the TC plus placebo and TC plus bevacizumab groups, respectively, indicating a significant prolongation with bevacizumab (HR = 0.717) [15].

In the ICON7 study, bevacizumab (7.5 mg/kg) was co-administered with TC therapy to 1528 patients with stage I–IV ovarian cancer for 6 cycles, followed by 12 cycles after completion of TC therapy, and this group was compared with the TC plus placebo group. The median PFS was 17.3 months and 19.0 months, in the TC plus placebo and TC plus bevacizumab groups, respectively, indicating a significant prolongation with bevacizumab (HR = 0.81) [16]. In summary, the addition of bevacizumab to the first-line chemotherapy significantly prolonged median PFS, but not OS. Presently, there are no reports of the prolongation of OS with the addition of bevacizumab. From the viewpoint of setting the goal of cancer treatment at OS prolongation, it is reasonable to consider that the add-on benefit of bevacizumab has not been established.

In addition, although the ROSiA study was a phase 2 study in patients with different characteristics, it demonstrated a further prolongation of PFS [20]. This was a phase 2 study of 1021 patients with stage IIB–IV epithelial ovarian, fallopian tube, or primary peritoneal cancer; grade 3 clear cell carcinoma, or stage I–IIA carcinosarcoma, where TC therapy was administered as the first-line chemotherapy for 4 to 8 cycles and bevacizumab was administered as maintenance therapy for up to 36 cycles to verify its safety and efficacy. The treatment consisted of paclitaxel 175 mg/m^2^ every 3 weeks or 80 mg/m^2^ weekly, carboplatin at a target area under the plasma concentration-time curve (AUC) of 5 or 6 every 3 weeks, and bevacizumab 15 mg/kg every 3 weeks or 7.5 mg/kg every 2 weeks. Grade 3 or higher adverse events were reported in 53.8% of all patients, including neutropenia (29.3%), febrile neutropenia (2.9%), hypertension (24.7%), thrombocytopenia (9.8%), proteinuria (3.8%), thrombosis (2.9%), and gastrointestinal perforation (1.4%). Interestingly, all of these adverse events occurred within the first 22 cycles of bevacizumab treatment and rarely occurred after that. The adverse events associated with long-term bevacizumab treatment were hypertension and proteinuria, which were rarely observed after cycle 23. The median PFS was 25.5 months, and the OS was not reached because 50% of the events did not occur. The median number of bevacizumab treatment cycles was 23, and the median duration of bevacizumab treatment was 15.5 months. In addition, 632 (62%), 537 (53%), and 298 (29%) of the patients received bevacizumab for ≥12 months, ≥15 months, and ≥24 months, respectively. Although there were differences in the patient characteristics, the number of cycles of the first-line chemotherapy, and the dosing methods in this study, the incidence of adverse events was comparable to that reported in other studies, and the median PFS was favorable. This clinical study reported the possibility of improving the prognosis of ovarian cancer by continuing maintenance therapy as long as possible instead of setting the number of doses of bevacizumab in advance.

Next is a brief introduction of the BOOST trial, which is currently ongoing. The subjects were patients with stage IIB–IV epithelial ovarian, fallopian tube, or primary peritoneal cancer. In this phase 3 clinical trial, patients are receiving paclitaxel 175 mg/m^2^ every 3 weeks and carboplatin AUC 5 every 3 weeks in combination with bevacizumab for 6 cycles. Bevacizumab 15 mg/kg is being administered every 3 weeks, and treatment will be administered for 22 cycles and 44 cycles in the control and experimental arms, respectively, and the PFS rates will be compared. The study commenced in November 2011 with a target sample size of 800, and enrollment was completed in November 2018 [21]. Because the 3-year observation period is planned after the end of enrollment, considerable time will be required for the results to be analyzed and reported. However, in this clinical study, the long-term continuation of maintenance therapy with bevacizumab may improve the prognosis of ovarian cancer.

### 3.2. Olaparib

In Japan, the PARP inhibitor, olaparib, was approved for coverage by health insurance as a maintenance therapy for platinum-sensitive recurrent ovarian cancer in April 2018. This was because of considerable evidence from the overseas Study 19 [22] and SOLO-2 studies [23]. Furthermore, maintenance therapy after the first-line chemotherapy was additionally approved for patients with germline BRCA-mutated (g*BRCA*m) cancer in June 2019. The SOLO-1 study verified the usefulness of maintenance therapy after the first-line chemotherapy. A total of 391 patients with stage III/IV tumor BRCA-mutated (t*BRCA*m) ovarian cancer showing platinum sensitivity in the first-line treatment were assigned to receive olaparib (300 mg × 2) orally (260 patients) or a placebo (131 patients). The median PFS rate was 56.8 months in the olaparib group and 13.8 months in the placebo group (HR, 0.33; 95% CI, 0.25–0.43; *p* < 0.0001), indicating a significant prolongation in the olaparib group compared to the placebo group [17].

In December 2020, cotreatment with bevacizumab was additionally approved as a maintenance therapy after the first-line chemotherapy in patients with homologous recombination deficiency (HRD). This is because approximately half of the patients with advanced ovarian cancer (FIGO stage III/IV) have HRD [24], and the usefulness of this agent was confirmed in a global phase 3 (PAOLA-1/ENGOT-ov25) study. This phase 3 study compared olaparib plus bevacizumab with bevacizumab alone as maintenance therapy in patients with advanced ovarian cancer who responded to the first-line treatment with platinum plus taxane plus bevacizumab. The study population consisted of 806 patients diagnosed with stage III/IV high-grade serous carcinoma, endometrioid ovarian carcinoma, fallopian tube cancer, or peritoneal cancer who received bevacizumab for 3 cycles or more in combination with platinum plus taxane-based chemotherapy and were determined to have achieved complete response (CR) or partial response (PR). The study group (537 patients) received olaparib (300 mg × 2/day) plus bevacizumab (15 mg/kg, every 3 weeks), and the control group (269 patients) received a placebo for Olaparib plus bevacizumab (15 mg/kg, every 3 weeks). The median PFS rates as the primary endpoint, was 22.1 months and 16.6 months in the study and control groups, respectively, indicating a significant prolongation in the study group compared to the control (HR, 0.59; 95% CI, 0.49–0.72; *p* < 0.0001). The median PFS rates were 37.2 months and 21.7 months in the study and control groups, respectively, (HR, 0.31; 95% CI, 0.20–0.47) for patients with *BRCA* mutations, and 18.9 months and 16.0 months in the study and control groups, respectively, (HR, 0.71; 95% CI, 0.58–0.88) for patients without *BRCA* mutations. The incidence of treatment-related adverse events was 99% and 96% in the study and control groups, respectively, and the incidence of ≥grade 3 treatment-related adverse events was 57% and 51% in the study and control groups, respectively. The most common events were fatigue/asthenia, nausea, and hypertension in the study group and hypertension, fatigue/asthenia, and arthralgia in the control group. Notably, the incidence of ≥grade 3 anemia was 17% and ≤1% in the study and control groups, respectively [18].

Although tests for g*BRCA* mutation and HRD are required when olaparib is administered as the first-line maintenance therapy, available treatment options have increased. While the first-line maintenance therapy with olaparib was shown to significantly prolong PFS, additional evidence is needed to verify its prolongation of OS.

### 3.3. Niraparib

Niraparib, which was approved in Japan in September 2020, is the second PARP inhibitor developed after olaparib. This agent acts selectively on cancer cells lacking homologous recombination repair, which is a DNA double-strand break repair mechanism, leading to cell death and antitumor effects. Olaparib was administered twice daily, whereas niraparib was administered once daily. Furthermore, olaparib is restricted to patients with a g*BRCA* mutation when administered as maintenance therapy after the first-line chemotherapy, whereas niraparib can be administered regardless of *BRCA* mutation status. The approval was based on the results of the PRIMA study [19] and the NOVA study [25], which were overseas phase 3 studies; the QUADRA study, [26] an overseas phase 2 study; the Niraparib-2001 study, [27] a Japanese phase 2 study examining safety in Japanese patients with ovarian cancer; and the Niraparib-2002 study [28], a Japanese phase 2 study examining efficacy and safety in Japanese patients with ovarian cancer. The PRIMA study is reviewed in this article.

In the PRIMA study, 733 patients with stage III/IV ovarian, fallopian tube, or primary peritoneal cancer who had achieved CR or PR following treatment with the platinum-based first-line chemotherapy were randomized to receive niraparib (300 mg/day) or a placebo in a 2:1 ratio, and the usefulness of this treatment was verified. PFS, the primary efficacy endpoint, was compared between the HRD-positive group and each of the other groups. The proportion of HRD-positive patients was 50.9%, and the median PFS in the HRD-positive group was 21.9 months (19.3 to upper limit unknown) in the niraparib group vs. 10.4 months (8.1 to 12.1 months) in the placebo group (HR, 0.43; 95% CI, 0.31–0.59; *p* < 0.0001). The median PFS rates were 13.8 months in the niraparib group vs. 8.2 months in the placebo group (HR, 0.62; 95% CI, 0.50–0.76; *p* < 0.0001). The most common ≥grade 3 adverse events reported in the niraparib group were anemia (31.0%), thrombocytopenia (28.7%), and neutropenia (12.8%) [19].

As described above, bevacizumab, olaparib, and niraparib can be used as a maintenance therapy after the first-line chemotherapy. However, there are safety concerns because of the limited data on olaparib and niraparib in Japanese patients. In addition, it may take some time to accumulate supporting evidence to determine which of these drugs should be used as the first-line drug.

### 3.4. Rucaparib

The approval status of PARP inhibitors in Japan and other countries is summarized in Table 4. Rucaparib is currently approved only for the treatment of recurrences in Europe and the US but has not been approved in Japan. The ongoing ATHENA study is focused on verifying the usefulness of maintenance therapy following the first-line chemotherapy. This is a phase 3 study to evaluate maintenance therapy with rucaparib in combination with nivolumab in new patients with advanced ovarian cancer who have responded to the first-line platinum-based chemotherapy. Patients are receiving oral rucaparib (600 mg twice daily) in combination with intravenous nivolumab (480 mg every 4 weeks). 

Patients were randomized to receive oral rucaparib 600 mg twice daily with intravenous nivolumab 480 mg every 4 weeks (arm A), oral rucaparib 600 mg twice daily with an intravenous placebo (nivolumab, arm B), an oral placebo (rucaparib) with intravenous nivolumab 480 mg every 4 weeks (arm C), or an oral placebo (rucaparib) with an intravenous placebo (nivolumab) every 4 weeks (arm D) in a 4:4:1:1 ratio, and PFS will be compared. A total of 1082 patients were enrolled, including 33 Japanese patients. The enrollment was completed in July 2020, and the analysis is currently underway [29].

## 4. Maintenance Therapy with Immune Checkpoint Inhibitors

Immune checkpoint inhibitors are anticancer drugs that target immune checkpoints, which suppress the action of immune cells. There are several types of immune checkpoints, and drugs targeting three immune checkpoint molecules—PD-1, PD-L1, and cytotoxic T-lymphocyte antigen 4 (CTLA-4)—have been approved in Japan, but not for ovarian cancer.

In the treatment of ovarian cancer, no recent evidence of breakthrough therapies that prolong OS has been provided. However, immune checkpoint inhibitors are attracting attention as drugs that can prolong OS. Several clinical studies of the usefulness of maintenance therapy after the first-line chemotherapy with immune checkpoint inhibitors have been completed or are ongoing. The following are clinical studies verifying the effect of adding immune checkpoint inhibitors to first-line chemotherapy. (Table 5).

### 4.1. Anti-PD-1 Antibody

#### 4.1.1. Nivolumab

Please refer to the aforementioned ATHENA study for this drug.

#### 4.1.2. Pembrolizumab

In the ongoing phase 3 (KEYLYNK-001/ENGOT-ov43) clinical study, patients with previously untreated, advanced ovarian cancer are receiving pembrolizumab in combination with standard chemotherapy, followed by maintenance therapy with olaparib [30]. In this study, 1086 patients without a somatic BRCA-mutation (s*BRCA*m) received 2 to 6 cycles of TC therapy in combination with pembrolizumab 200 mg, followed by maintenance therapy with pembrolizumab 200 mg plus olaparib 600 mg, pembrolizumab 200 mg plus a placebo for olaparib, or a placebo for pembrolizumab plus a placebo for olaparib. The PFS and OS will be compared and treatment is planned to be administered for up to 35 cycles of 3 weeks each.

### 4.2. Anti-PD-L1 Antibody

#### 4.2.1. Avelumab

Avelumab, a human anti-PD-L1 antibody, was evaluated in the first phase 3 study of combination therapy with chemotherapy for ovarian cancer. The JAVELIN Ovarian 100 study was a global phase 3 study of 998 patients with previously untreated, locally advanced/metastatic ovarian cancer who were randomized to receive carboplatin with paclitaxel followed by observation (arm A), carboplatin with paclitaxel followed by maintenance therapy with avelumab alone (arm B), or carboplatin with paclitaxel and avelumab followed by maintenance therapy with avelumab alone (arm C). The superiority of PFS in arms B and C, which received avelumab, over arm A that did not receive avelumab was investigated as the primary endpoint. However, an interim analysis did not show any add-on effect of avelumab alone or in combination with chemotherapy in the standard treatment, resulting in early discontinuation [33].

Subsequently, a global phase 3 study was conducted in 700 patients with previously untreated, advanced ovarian cancer (JAVELIN Ovarian PARP 100, JapicCTI-194567) [34]. The study included three arms: platinum chemotherapy in combination with avelumab followed by maintenance therapy with avelumab and talazoparib, platinum chemotherapy followed by maintenance therapy with talazoparib, and platinum chemotherapy in combination with bevacizumab followed by maintenance therapy with bevacizumab. The primary endpoint was PFS, as assessed using response evaluation criteria in solid tumors version 1.1 (RECIST v1.1), and Japanese institutions participated in the study. However, this study was terminated early, mainly because the extent of the benefit observed with avelumab in the initial treatment of the unselected population did not support the study continuation. This finding indicated the need for a better understanding of the role of immunotherapy in ovarian cancer. In addition, the treatment strategy for ovarian cancer is changing rapidly, with the approval of a PARP inhibitor as the first-line maintenance therapy. It has been reported that the study was not terminated for safety reasons [35].

#### 4.2.2. Durvalumab

Durvalumab, a humanized anti-PD-L1 antibody, has shown usefulness in phase 1 studies as a combination therapy with olaparib or cediranib for recurrent ovarian cancer and is expected to have the potential for maintenance therapy. The DUO-O study has been underway since 2019. This phase 3 study will evaluate the efficacy and safety of standard platinum-based chemotherapy in combination with durvalumab and bevacizumab followed by maintenance bevacizumab alone, maintenance durvalumab with bevacizumab, or maintenance durvalumab with bevacizumab and olaparib in 1254 patients with stage III/IV epithelial ovarian cancer [31]. Patient enrollment will continue until 2023.

#### 4.2.3. Atezolizumab

Atezolizumab is a humanized anti-PD-L1 antibody. The IMagyn050 study was a randomized controlled study of four drugs that compared atezolizumab with a placebo in patients with previously untreated ovarian cancer receiving paclitaxel, carboplatin, and bevacizumab [32]. A total of 1301 patients were randomized to receive either atezolizumab in combination with paclitaxel, carboplatin, and bevacizumab or a placebo in combination with paclitaxel, carboplatin, and bevacizumab in a 1:1 ratio. However, at the European Society for Medical Oncology (ESMO) Congress in 2020, the median PFS rates were 19.5 months and 18.4 months in the atezolizumab and placebo groups, respectively, showing no statistically significant prolongation of PFS, the primary endpoint [36]. For the other primary endpoint of OS, follow-up will continue until the next scheduled analysis timepoint.

Table 5 shows the current clinical studies described above. Immune checkpoint inhibitors are rapidly becoming a new treatment option for cancer. However, the response rate to immune checkpoint inhibitors alone for solid cancers varies according to the type of cancer, and the response has been modest for ovarian cancer based on previous reports. Therefore, there are expectations for the combination therapy regimens described in this article, and the results of large-scale phase 3 studies will become available in the coming years. In contrast, there are reports of immune-related adverse events that have not been observed by obstetricians, indicating the need for new safety management. In addition, biomarkers for immune checkpoint inhibitors have not been identified, which is an ongoing challenge.

## 5. Conclusions

The first-line treatment for advanced ovarian cancer has changed significantly over the past few years. Currently, attention is focused on maintenance therapy, which was previously considered ineffective. The prognosis is expected to improve with the use of molecular-targeted drugs and immune checkpoint inhibitors as maintenance therapy. Although biomarkers have been identified for each molecular-targeted drug, there are none for immune checkpoint inhibitors. The development of biomarkers for immune checkpoint inhibitors is an ongoing challenge that needs to be addressed in the future. Further evidence is needed to improve the prognosis of ovarian cancer.

## Figures and Tables

**Table 1:** Previous trials of first maintenance therapy using cytotoxic agent.

Trials	Patients	*N*	Arms	Median PFS	Hazard Ratio/*p* Value	Median OS	Hazard Ratio/*p* Value
GOG178 [5]	Stage III–IV	277	(1) PTX * × 3	14	*p* = 0.006	48	*p* = 0.34
			(2) PTX * × 12	22		53	
AGO-GINECO [6]	Stage IIB–IV	1308	(1) TC × 6→observation	18.5	HR:0.97 *p* = 0.688	43.1	HR:1.01 *p* = 0.885
			(2) TC × 6→NGT × 4	18.2		44.5	
MITO-1 [7]	Stage IC–IV	273	(1) TC × 6→observation	28.4	HR:1.18 *p* = 0.83	NA	NA
			(2) TC × 6→NGT × 4	18.2		NA	
After-6 [8]	Stage III–IV	200	(1) TC × 6→observation	30	*p* = 0.68	NR	*p* = 0.13
			(2) TC × 6→PTX ** × 6	34		77	

Abbreviations: PTX *, paclitaxel 135 mg/m^2^ on day 1, every 4 weeks; PTX **, paclitaxel 175 mg/m^2^ on day 1, every 3 weeks; NGT, Nogitecan 1.5 mg/m^2^ on days 1 through 5, four cycles, every 3 weeks; TC, paclitaxel + carboplatin; PFS, progression-free survival; HR, hazard ratio; OS, overall survival; NA, not available; NR, not reached.

**Table 2:** Clinical trials of primary therapy for ovarian cancer.

Trials	Patients	*N*	Arms	Median PFS (Months)	HR (95% CI)/IQR *p* Value
JGOG3016 [10]	Stage II–IV	631	(1) TC × 6–9	17.2	0.71(0.58–0.88) *p* = 0.0015
			(2) dose-dense TC × 6–9	28.0	
GOG262 [12]	incompletely resected stage II/III Stage IV	692	(1) TC(±Bev) × 6→(±Bev)	14.0	0.89 (0.74–1.06) *p* = 0.18
			(2) dose-dense TC (±Bev) × 6→(±Bev)	14.7	
ICON8 [13]	Stage IC–IV High-risk IC/IIA	1566	(1) TC × 6	17.7	IQR (10.6-NR)
			(2) dose-dense TC × 6	20.8	IQR (11.9–59.0): *p* = 0.35
			(3) Weekly TC × 6	21.0	IQR (21.0–54.0): *p* = 0.51

Abbreviations: TC, paclitaxel + carboplatin: PFS, progression-free survival; Bev, bevacizumab; HR, hazard ratio; 95% CI, 95% confidence interval; IQR, interquartile range.

**Table 3:** Previous clinical trials of maintenance therapy.

Trials	Patients	*N*	Arm	Median PFS (Month)	PFS HR (95% CI): *p* Value
GOG218 [15]	Stage III with any gross residual disease Stage IV	1800	(1) TC→TC + Placebo × 5→Placebo × 16	10.3	-
			(2) TC + TC + Bev × 5→Placebo × 16	11.2	0.908 (0.795–1.040): *p* = 0.16
			(3) TC + TC + Bev × 5→Bev × 16	14.1	0.717(0.625–0.824): *p* < 0.001
ICON7 [16]	High-risk early stage Stage IIB–IV	1528	(1) TC × 6	17.3	0.81(0.70–0.94): *p* = 0.0041
			(2) TC→TC + Bev × 5→Bev × 12	19.0	
SOLO-1 [17]	Stage III–IV HGSC or EM g/s*BRCA*m	391	(1) Platinum-based × 6–9→ Placebo	13.8	0.33 (0.25–0.43) *p* < 0.0001
			(2) Platinum-based × 6–9→ Olaparib	56.8	
PAOLA-1 [18]	Stage III–IV HGSC	806	(1) Platinum/taxane/Bev→Bev	16.6	0.59 (0.49–0.72): *p* < 0.0001
			(2) Platinum/taxane/Bev→Bev/olaparib	22.1	
PRIMA [19]	Stage III–IV HGSC or EM non-mucinous *BRCA*m	733	(1) Platinum-based × 6–9→ Placebo	8.2	0.62 (0.50–0.76): *p* < 0.001
			(2) Platinum-based × 6–9→ Niraparib	13.8	

Abbreviations: g/s*BRCA*m, germline or somatic *BRCA* mutation; HGSC, high-grade serous carcinoma; EM, endometrioid carcinoma; TC, paclitaxel + carboplatin; Bev, bevacizumab; PFS, progression-free survival; NR, not reached; HR, hazard ratio; 95% CI, 95% confidence interval.

**Table 4:** Indication of PARP inhibitors for patients with epithelial ovarian cancer.

Drug	Agency	Indications	*BRCA*/HRD Status	Clinical Setting	Dosing
Olaparib	FDA	Advanced EOC, post CR/PR	g/s*BRCA*	First-line Maintenance	300 mg BID
		Advanced EOC	g*BRCA*	Monotherapy, Fourth-line	
		Platinum-sensitive recurrent OC, post CR/PR	-	Maintenance	
	EMA	Advanced EOC, post CR/PR	g*BRCA*	Maintenance	
		Platinum-sensitive recurrent HGOC, post CR/PR	-		
	JPN	Platinum-sensitive recurrent, post CR/PR	-	Maintenance	
		Advanced OC	g*BRCA*	Maintenance	
		Advanced OC, with bevacizumab	HRD	Maintenance	
Rucaparib	FDA	Advanced OC	g/s*BRCA*	Monotherapy, Third-line	600 mg BID
		Platinum-sensitive recurrent OC, post CR/PR	-	Maintenance	
	EMA	Platinum-sensitive recurrent or progressive HGOC	g/s*BRCA*	Monotherapy, Third-line	
		Platinum-sensitive recurrent OC, post CR/PR	-	Maintenance	
	JPN	Not approved			
Niraparib	FDA	Advanced OC, platinum-sensitive recurrent OC, post CR/PR	-	Maintenance	200 or 300 mg QD
		Platinum-sensitive recurrent	HRD	Monotherapy, Fourth-line	
	EMA	Advanced OC, HGOC, post CR/PR	-	Maintenance	
		Platinum-sensitive recurrent, HGSOC, post CR/PR		Maintenance	
	JPN	Advanced OC or platinum-sensitive recurrent, post CR/PR	-	Maintenance	
		Platinum-sensitive recurrent	HRD	Monotherapy, Fourth-line	

Abbreviations: FDA, Food and Drug Administration; EMA, European Medicine Agency; JPN, Japan; Post CR/PR, post complete or partial response to platinum-based chemotherapy; HGOC, high-grade epithelial ovarian, fallopian tube, or primary peritoneal cancer; HGSOC, high-grade serous epithelial ovarian, fallopian tube, or primary peritoneal cancer; OC, epithelial ovarian, fallopian tube, or primary peritoneal cancer; g/s*BRCA*, germline and/or somatic *BRCA* mutation; HRD, homologous recombination deficiency; BID, twice daily; QD, once daily.

**Table 5:** Ongoing clinical trials of first maintenance therapy using checkpoint inhibitor.

Trials	Patients	*N*	Arms	Primary Endopoint
ATHENA [29]	Stage III–IV HGSC Post CR/PR to platinum-based chemotherapy	1082	(1) Rucaparib + Niborumab	PFS
			(2) Rucaparib + Placebo	
			(3) Placebo + Niborumab	
			(4) Placebo + Placebo	
KEYLYNK-001/ENGOT-ov43 [30]	Stage IIB–IV s*BRCA*m(-)	1086	(1) TC→TC + Pembrolizumab × 5→Pembrolizumab + Olaparib	PFS OS
			(2) TC→TC + Pembrolizumab × 5→Pembrolizumab + Placebo	
			(3) TC→TC + Pembrolizumab × 5→Placebo + Placebo	
DUO-O [31]	Stage III–IV s*BRCA*m(-)	1254	(1) Platinum-based chemotherapy + Bev + placebo → Bev + placebo + placebo	PFS
			(2) Platinum-based chemotherapy + Bev + durvalumab → Bev + durvalumab + placebo	
			(3) Platinum-based chemotherapy + BEV + durvalumab → Bev + durvalumab + olaparib	
IMaGYN050 [32]	Stage III–IV	1301	(1) TC + Bev+ Atezolizumab→Bev + Atezolizumab	PFS OS
			(2) TC + Bev+ Placebo→Bev + Placebo	

Abbreviations: CR, complete response; PR, pertchial response; HGSC, high-grade serous carcinoma; s*BRCA*m, somatic *BRCA* mutation; TC, paclitaxel + carboplatin; Bev, bevacizumab; PFS, progression-free survival; OS, overall survival.

## Data Availability

Not applicable.
